# Longitudinal surveillance of *Coxiella burnetii* following an abortion storm in domestic goats

**DOI:** 10.3389/fvets.2024.1426573

**Published:** 2024-09-13

**Authors:** Halie K. Miller, Rachael A. Priestley, Cody B. Smith, Cara Cherry, Gilbert J. Kersh

**Affiliations:** Rickettsial Zoonoses Branch, Centers for Disease Control and Prevention, Atlanta, GA, United States

**Keywords:** coxiellosis, one health, Q fever, zoonosis, livestock

## Abstract

Q fever is a disease caused by *Coxiella burnetii*, which can cause serious illness in humans and abortions in goats. A Q fever outbreak among an unvaccinated goat herd led to a 65% loss of the kid crop in spring 2018. To assess the impact of the outbreak on the herd and environment, longitudinal surveillance of the ranch was conducted across three samplings in September 2018, April 2019, and May 2022. Antibodies against *C. burnetii* were monitored by an indirect immunofluorescence assay. Shedding was monitored through analysis of vaginal/fecal swabs and milk. Environmental swabs and bulk soil were collected from various locations around the ranch. Animal and environmental samples were analyzed for *C. burnetii* DNA by PCR. Herd-level seroprevalence decreased from 89% in 2018 to 84.3% in 2019, and 64.5% in 2022. Overall herd shedding was 14.4% in 2018, 7.4% in 2019, and 6.7% in 2022. The percentage of *C. burnetii-positive* environmental samples was 83.7% in 2018, 51.7% in 2019, and 28.6% in 2022. Serological evidence suggests that new infections were occurring in the herd 4 years post-abortion storm. This study demonstrates the presence of *C. burnetii* shedding and environmental contamination in a goat operation at least four kidding seasons after an outbreak. A better understanding of management practices that can improve outcomes for infected herds, particularly in areas without access to vaccines against *C. burnetii*, is needed to better protect operators and the public.

## Introduction

Abortion storms are often the first symptom of a *C. burnetii*-infected goat herd as these important reservoir hosts may show no other overt signs of illness. *C. burnetii*-dependent abortion storms are characterized by late-term abortions, stillbirths, and weak offspring, which may affect more than 50% of the herd (1). This zoonotic bacterial pathogen is shed in urine, feces, vaginal secretions, milk, and parturition byproducts. *C. burnetii* aerosolized from these excretion routes can be transmitted to humans through inhalation, particularly during abortion events due to massive shedding during parturition. Placentas have been reported to contain as much as 10^9^ *C. burnetii* per gram of tissue, and shedding can occur following live births as well (2–4). Inhalation of fewer than 10 organisms has been demonstrated as sufficient to cause human infection (5).

*C. burnetii* is the causative agent of Q fever disease in humans, and the major route of infection is through inhalation (6, 7). Acute illness may be asymptomatic or self-limiting; however, severe pneumonia or hepatitis is possible. Chronic Q fever is a severe form of the disease characterized most often by life-threatening endocarditis, which can develop years after the infection. The role of goats in the transmission of *C. burnetii* to humans has been well documented. The historic Netherlands outbreak in 2007 led to over 4,000 symptomatic human cases and an estimated 40,000 exposures linked to *C. burnetii*-infected dairy goat and sheep herds (8, 9). A serosurvey in Michigan found goat owners were almost three times more likely to have antibodies against *C. burnetii* (10). Goat-associated outbreaks in the United States have occurred in Colorado, Washington, and Montana, resulting in cases of human Q fever (11, 12).

*C. burnetii* is adept at persisting in the environment, which presents a considerable public health concern following a massive shedding event, such as an outbreak in a goat herd. Few studies have characterized the long-term consequences of *C. burnetii* infection in naturally infected herds, and the majority of available studies have focused on the effect of vaccination in dairy goats (13–18). Despite receiving annual vaccination, dairy goats have been shown to shed *C. burnetii* over three kidding seasons and viable *C. burnetii* has been detected in the farm environment until the third kidding season post-outbreak (14, 19). A recent study of vaccinated dairy goats found shedding was highest within the first three kidding seasons post-outbreak, yet *C. burnetii* shedding of the herd continued until the seventh kidding season (19). *C. burnetii* shedding in unvaccinated dairy goat herds has been documented for at least 2 years (16, 20). Infection of the placenta in unvaccinated goats was limited to the second kidding season post-outbreak (17). Long-term surveillance data of goat herds beyond two successive kidding seasons are limited, particularly among unvaccinated, non-dairy herds. Livestock vaccination, believed to be the best Q fever prevention and control strategy, is not broadly applicable to countries such as the United States for which no Q fever vaccine is licensed (15, 18, 21–29). A recent study of domestic doe goats across the United States demonstrated that 7.8% of operations were positive for the shedding of *C. burnetii* in vaginal secretions and no significant difference was found by primary production of the operation (30). A model of *C. burnetii* transmission in goats identified 6 years of vaccination as the most effective strategy for controlling an outbreak (15). In the absence of vaccination or other control measures, the model predicted persistence for upward of 10 years (15). Given the sporadic nature of *C. burnetii* outbreaks and the devastating public health and economic repercussions, additional epidemiological data from natural outbreaks are essential. A *C. burnetii* outbreak among a herd of non-dairy goats in the United States provided the opportunity to investigate the persistence, infection dynamics, and environmental contamination in the absence of vaccination. This study was conducted over a 4-year period (2018–2022) with the goal of expanding the current understanding of the longitudinal consequences of *C. burnetii* infection in goats. To this end, we conducted an observational study to determine whether *C. burnetii* within the herd and environment remain during subsequent parturitions in a non-dairy operation in the absence of vaccines.

## Methods

### Ethical statement

All procedures contributing to this work comply with the ethical standards of the relevant national and institutional guides on the use of animals in research. All animal experiments were performed according to an animal protocol approved by the Centers for Disease Control and Prevention (CDC) Atlanta Institutional Animal Care and Use Committee (IACUC). Where applicable, this study is reported in accordance with the recommendations in the ARRIVE guidelines. To reduce the risk of human illness and bacterial spread, personal protective equipment (PPE) consisting of a Tyvek protective suit, gloves, N95 respirators, and rubber boots were utilized during all sampling periods. Personnel were fit tested for N95 respirator protection. PPE was disinfected by autoclaving or treatment with 5% Microchem.

### Herd history and outbreak description

**Figure 1:**
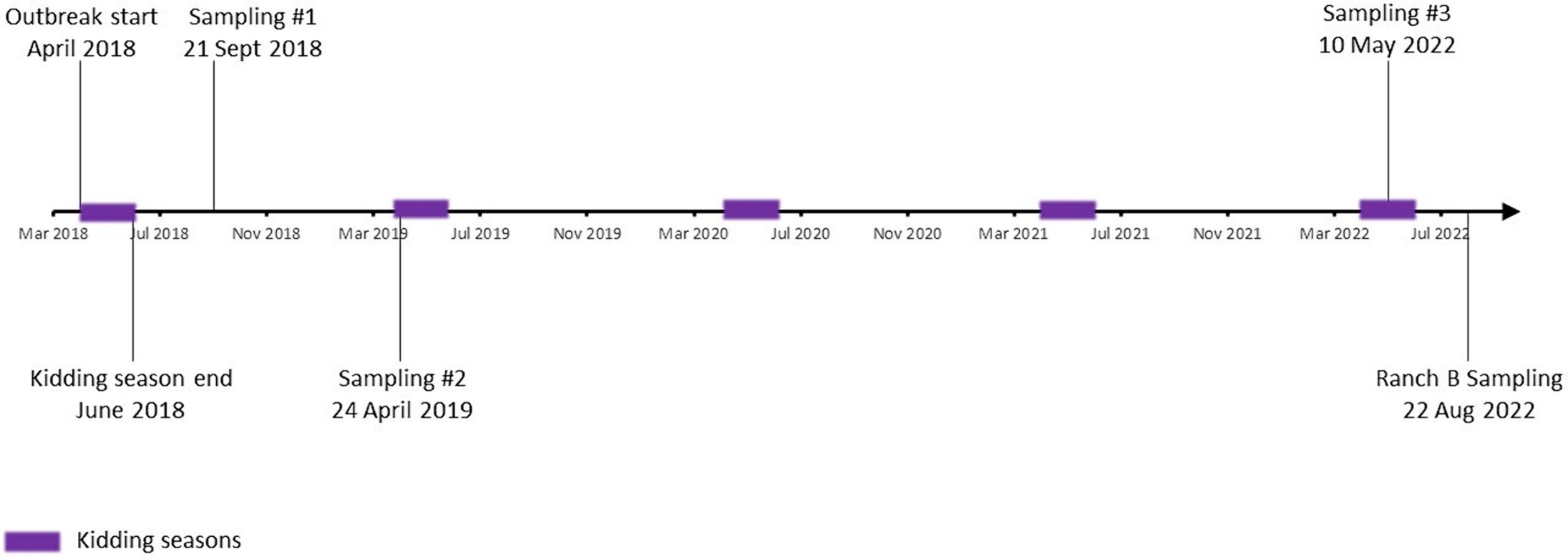
Study timeline. Timeframe from the initial abortion event marking the beginning of the outbreak in 2018 through the final sampling in 2022 is depicted. Kidding seasons are noted as purple squares.

The main study site, ranch A, is a privately owned facility that experienced an abortion storm from April to June 2018 (Figure 1). During that time, the goat herd was approximately 125–150 head and 65.6% of the kid crop were lost to abortions. Abortions occurred ~10–14 days preterm and manifested as deceased fetuses encompassed in thickened purulent placentas with white plaques. Fetal and placental samples submitted for abortion screen testing to a veterinary diagnostic laboratory indicated *C. burnetii* as the causative agent. Interestingly, 15% of the does were lost due to post-kidding complications, namely severe mastitis and septicemia. Metritis has been described as a symptom of *C. burnetii* infection in dairy goat does; however, evidence of direct causation is lacking (31). Morbidity and mortality in does of this magnitude are not often reported for *C. burnetii*, as such the presence of additional etiologies cannot be ruled out. At the discretion of the operator and the attending veterinarian, oxytetracycline was given by injection to all pregnant does every 2–3 days until kidding. Additionally, the herd was placed on a treatment-level dose of chlortetracycline (CTC) in the feed, which was continued during subsequent birthing seasons through 2021. Biosecurity practices on the operation included disposal of placentas by burning and limiting visitors to the operation. The operator reported that four persons associated with the ranch had positive antibody titers, one of which had also reported symptoms compatible with acute Q fever. The herd was otherwise healthy and experienced no further abortions until one in 2022 from an unknown cause. It was not attributed to *C. burnetii* based on molecular diagnostic testing of the placenta conducted at a veterinary diagnostic laboratory. Based on testing conducted herein, the doe was also negative for *C. burnetii* in milk and vaginal secretions. The herd was mainly comprised of Boers bred for show and were largely kept outdoors with access to quonset shelters and pasture for grazing. Due to factors unrelated to the outbreak, the operator reduced the size of the herd by ~78% by 2022. Goats remaining in 2022 were chosen based solely on quality and genetics.

### Sample procurement

Sample collection occurred in September 2018, April 2019, and May 2022 (Figure 1). In 2018, ~83.3% (125/150) of the herd had at least one specimen analyzed (Table 1; Supplementary Table S1). Goats were selected for inclusion at the operator’s discretion; however, sampling was representative of the composition of the herd at the time with regard to sex, age, and reproductive status. In 2019 and 2022, 100% of the herd was tested (136 and 33 goats, respectively). In August 2022, a single sampling occurred at a secondary operation (ranch B) whose primary production purpose is raising goats for show. Ranch B is a privately owned facility that purchased ~25% of the goats from ranch A between 2018 and 2022. The 2022 sampling of ranch B included four doe goats that had been purchased after the 2019 sampling of ranch A. No confirmed uninfected herds were available to serve as a control group. Randomization, blinding, and controlling for cofounders were not possible.

**Table 1:** Samples by type and year.

	2018		2019		2022	
Sample type	Total *n*	Positive *n* (%)	Total *n*	Positive *n* (%)	Total *n*	Positive *n* (%)
Animal						
Serology						
Total seropositive*	100	89 (89.0)	134	113 (84.3)	31	20 (64.5)
PhII>PhI among seropositive goats	89	10 (11.2)	113	6 (5.3)	20	18 (90.0)
PCR						
Vaginal swabs	106	14 (13.2)	119	3 (2.5)	28	0 (0.0)
Fecal swabs	19	4 (21.1)	17	1 (5.9)	2	0 (0.0)
Milk	0	—	6	6 (100.0)	4	2 (50.0)
Total goats by PCR	125	18 (14.4)	136	10 (7.4)	30	2 (6.7)
Total goats any method	125 (83.3^‡^)	93 (74.4)	136 (100^‡^)	115 (83.9)	33 (100^‡^)	20 (60.6)
Environmental						
Bulk						
Kidding/nursery area	16	16 (100.0)	6	3 (50.0)	9	6 (66.7)
Goat-accessible area	4	4 (100.0)	6	3 (50.0)	5	1 (20.0)
Burn pit	4	3 (75.0)	1	1 (100.0)	1	0 (0.0)
Ranch equipment	1	1 (100.0)	0	–	0	–
Goat-inaccessible area	3	3 (100.0)	4	0 (0.0)	8	1 (12.5)
Total	28	27 (96.4)	17	7 (41.2)	23	8 (34.7)
Swab						
Kidding/nursery area	10	8 (80.0)	2	1 (50.0)	2	0 (0.0)
Goat-accessible area	1	0 (0.0)	0	–	0	–
Ranch equipment	4	1 (25.0)	6	6 (100.0)	2	0 (0.0)
Goat-inaccessible area	0	–	4	1 (25.0)	1	0 (0.0)
Total	15	9 (60.0)	12	8 (66.7)	5	0 (0.0)
Total by any sample type	43	36 (83.7)	29	15 (51.7)	28	8 (28.6)

*Titers ≥ 1:128 for either phase I or phase II were considered positive.

^‡Total percentage of the herd that was tested by any method based on estimated herd size.^

Goats were tested by either vaginal (does) or fecal (bucks/wethers/kids) swab. When available, milk was expressed from lactating does into 50-ml conical tubes. Dairy was not the primary production purpose of the operation; therefore, no milk samples were collected in 2018 as the kids had been weaned prior to the sampling period. Serum was collected via jugular venipuncture in vacutainer serum separator tubes (Becton Dickinson) and allowed to clot before centrifugation at the sample collection site. Swabs (vaginal/fecal/environmental) were sterile media-free rayon (BD). Vaginal swabs were collected as previously described (30). Fecal swabs were collected by gently rotating the swab 180 degrees in the rectum, 4–5 times. Environmental samples were collected as described previously (32). Briefly, environmental swabs from farm equipment (utility task vehicles, all-terrain vehicles, lawnmowers, and tractors), gates, fences, and feeders were collected by wiping the dry swab across a solid surface. Farm equipment was not cleaned or disinfected immediately prior to sampling; rather, sampling was conducted to reflect standard operating conditions. All swabs were processed as individual samples. Bulk sampling was conducted by collecting material from the ground with 50-ml conical tubes, which was typically the top layer of soil. For samples from fixed locations, hand-held global positioning satellite (GPS) units were utilized to collect sampling locations. All environmental samples were categorized as originating from either kidding/nursery areas, goat-accessible areas (barn, pens, pasture, pathways, excluding kidding nursing areas), goat-inaccessible area (representing areas of dispersal via environmental factors, equipment, workers, visitors, or other non-goat animals such as dogs or wildlife), a burn pit utilized for placenta disposal, or farm equipment. All samples were maintained at 4°C and shipped overnight to the laboratory on ice packs for processing. Sampling techniques were consistent across all study periods.

### Analysis of anti-*C. burnetii* antibodies from sera

IgG antibodies against *C. burnetii* were determined by immunofluorescence analysis (IFA) using an in-house assay as previously described (33). Briefly, slides were coated with standard diagnostic antigens, the Nine Mile phase I (PhI) (NMI) reference strain of *C. burnetii*, and Nine Mile phase II (PhII), an avirulent, laboratory-generated strain created from serial passage of NMI. During the course of infection, antibodies that recognize the PhII antigen typically develop early followed by PhI-specific antibodies later on. PhII-specific antibodies tend to decrease before PhI antibodies which typically remain over a longer period (17, 34–36). Slides were incubated with serum titrations and treated with a fluorescein isothiocyanate-conjugated rabbit anti-goat antibody. Antibody binding was determined via a fluorescence microscope. Titers against PhI or PhII ≥128 were considered positive. The geometric mean titer (GMT) was calculated by raising 2 to the power of the arithmetic mean of transformed titers (log base 2) as previously described (37). Titers <16 are not included in GMT calculations.

### Processing of milk samples

Milk was centrifuged at 1700xg for 15 min, and the supernatant/lipid layer was removed. Pellets were resuspended in 10–30 mL of PBS and centrifuged at 1700xg for 15 min. Supernatants/remaining lipids were removed, and pellets were resuspended in 1 mL PBS. A 500 μL aliquot was centrifuged at 16,000xg for 5 min, and the pellet was resuspended in 200 μL PBS for DNA extraction.

### Processing of swabs and bulk environmental samples

Swabs were vortexed in 800 μL of PBS for 30–60 s, followed by incubation at 35°C with shaking at 200 rpm for 1 h. DNA was extracted from a 200 μL aliquot. Bulk soil was processed as described previously with some exceptions (38). Briefly, 5 g of soil was incubated with 10-30 mL of PBS on an end-over-end rocker at room temperature for 1 h. Sediment was removed by centrifugation at 125xg for 5 min. The supernatant was centrifuged at 20,000xg for 15 min to concentrate microorganisms. Pellets were resuspended in 500 μL PBS, and DNA was extracted from a 200 μL aliquot.

### DNA extraction

Both animal (vaginal/fecal swabs and milk) and environmental (bulk soil and swabs) samples were processed using commercially available kits (Supplementary Table S2). Due to the discontinuation of kits by manufacturers, extraction kits differed across the study period (Supplementary Table S2). No significant difference in total DNA yield or purity (A260/A280 ratios) was noted for any kit based on spectrophotometer readings of representative samples (data not shown). Extractions were conducted per the manufacturer’s instructions.

### Real-time PCR analysis of *C. burnetii* DNA

Eluates were analyzed for *C. burnetii* DNA using an in-house quantitative TaqMan PCR assay specific for the multi-copy *IS1111* gene sequence as previously described (32). Eluates were spiked with a known quantity of *C. burnetii* DNA to test for PCR inhibitors as previously described (32). Samples displaying inhibition, as indicated by an increase of at least one cycle, were cleaned via the DNeasy PowerClean Pro Cleanup Kit (Qiagen) and reanalyzed. Ct >40 was considered negative. Genomic equivalents (GE) were calculated as previously described (38). Briefly, the single copy gene target, *com1*, was used to compare to the multi-copy *IS1111* Ct values. These were compared to standard curves generated from NMI, which harbors approximately 20 copies of the *IS1111* sequence. The outbreak strain was determined to have ~52 copies of the *IS1111* sequence based on three 2018 samples. This was used to approximate *C. burnetii* quantity across all samples. The presence of other strains on the ranch with different *IS1111* copy numbers may influence quantity estimates. Data are displayed as the geometric mean and 95% confidence interval of the geometric mean (CI) of log10-transformed quantity estimates (positive values only).

### Genotyping of *C. burnetii* DNA

Over 70 different genotypes of *C. burnetii* have been described based on multispacer sequence typing (MST) data (39). Three genotypes are routinely identified in the United States, sequence type 8 (ST8) (closely associated with goats), ST20 (closely associated with dairy cattle), and ST16/26 (no known associations) (11, 40–42). Genotyping was conducted on the sample with the greatest quantity of *C. burnetii* DNA, which was a bulk soil sample collected from a birthing pen in 2018 with an estimated quantity of 2.61×10^6 (Ct 18.0) from 2018. Genotyping was performed using a rapid PCR-based method to identify single nucleotide polymorphisms (SNP). This allows for the identification of the sequence type as defined by multispacer sequence typing as previously described (43).

### Statistical analysis

Differences in proportions were assessed by Fisher’s exact test with a Bonferroni *post-hoc* test for multiple comparisons where appropriate. These data were analyzed using RStudio v2023.12.1 (44). Welch’s one-way ANOVA with Dunnett’s T3 multiple comparisons test was used to determine the significance of transformed antibody titers across the study period. A paired *t*-test was used to determine the significance of serial antibody titers obtained from the same goats compared across two sampling periods. These data were analyzed using GraphPad Prism 10.2.2 (GraphPad (45)). *p* < 0.05 was deemed significant. *Post-hoc* analysis of achieved power based on the actual sample size and observed effect size, and an alpha of 0.05 was conducted using G*Power 3.1.9.7 (46).

## Results

### Shedding of *C. burnetii* following an abortion storm in a non-dairy goat herd

In 2018, 125 goats were tested for the shedding of *C. burnetii* by vaginal (does) or fecal (bucks/wethers/kids) swab (Table 1; Supplementary Table S1). Among vaginal swabs, 13.2% (14/106) were positive for *C. burnetii* DNA by IS1111 PCR while 21.1% (4/19) of fecal swabs were positive. The geometric mean (GM) quantity of *C. burnetii* across all positive swabs in 2018 was 59.8 (95% CI, 25.6–139.7) GE/swab. No milk was collected in 2018. Total herd-level shedding was 14.4% (18/125) in 2018. In 2019, 136 vaginal/fecal swabs were tested with *C. burnetii* DNA detected in 2.5% (3/119) of vaginal swabs and 5.9% (1/17) of fecal swabs. The GM quantity of *C. burnetii* among positive swabs in 2019 was 51.6 (95% CI, 15.0–177.3) GE/swab. All six milk samples from 2019 were positive with a GM quantity of 16.7 (95% CI, 7.7–36.1) GE/ml. Secretion of *C. burnetii* in the vaginal mucus was not detected in any of the six lactating does (Supplementary Table S1). Total shedding by any method in 2019 was 7.4% (10/136) (*p* = 0.22; power = 0.491, actual alpha = 0.029, relative to 2018). In 2022, *C. burnetii* was not detected in any vaginal (0/29) or fecal (0/1) swabs. Among the nursing does, 50.0% (2/4) were shedding *C. burnetii* in the milk with a GM quantity of 2.1 (95% CI, 0.05–92.7) GE/mL. Herd-level shedding in 2022 was 6.7% (2/30) (*p* = 1.00; power = 0.184, actual alpha = 0.005, relative to 2018) (*p* = 1.00; power = 0.009, actual alpha = 0.005, relative to 2019). Based on the small observed effect size coupled with the limited herd size, the achieved power was too low (<80%) to draw meaningful conclusions, regarding differences in the proportion of shedders across the sampling periods.

Management practices of ranch A limited the number of goats that were available for serial testing; however, 78 goats were tested for shedding of *C. burnetii* in both 2018 and 2019 (Supplementary Table S1). In total, 88.5% (69/78) were negative at both samplings. Seven goats were shedding in 2018 and two in 2019; however, no goats tested were shedding at both samplings (Supplementary Table S1). None of the goats from 2018 remained in the herd by 2022; however, testing of goats that had been purchased by a second ranch, ranch B, allowed for serial testing of four goats across all sampling periods; one was positive in 2018 (20.4 GE/swab), and none were positive in 2019 or 2022 (Supplementary Table S1).

### Serological analysis following a Q fever outbreak in goats reveals persistent titers across a 4-year period

**Figure 2:**
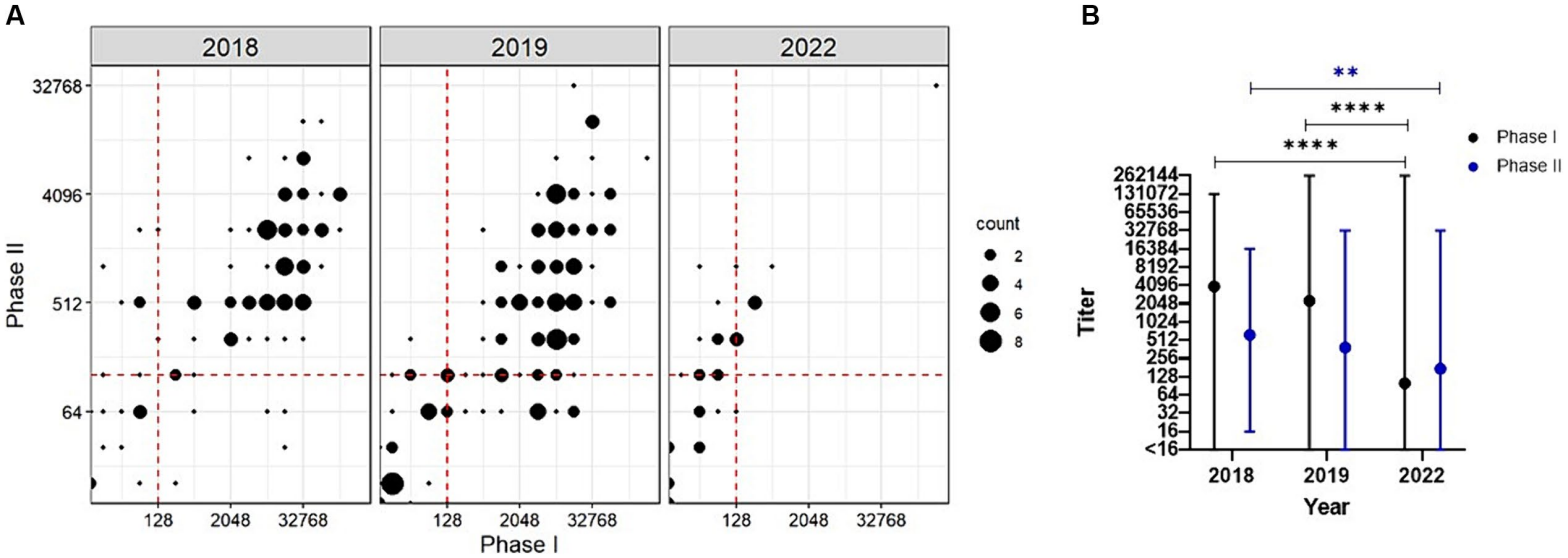
Serological analysis of goats following a *C. burnetii* abortion event. **(A)** Antibody titers against PhI *C. burnetii* are plotted against the PhII titers for each sampling year. Titers ≥128 are considered positive. This cutoff is indicated as a red dashed line. **(B)** GMT ± range is given for PhI (black) and PhII (blue) across the study period. **p < 0.01, ****p < 0.0001 as determined by Welch’s one-way ANOVA with Dunnett’s T3 multiple comparisons test.

In 2018, 89.0% (89/100) of the tested goats were positive for anti-*C. burnetii* IgG antibodies (Table 1; Supplementary Table S1; Figure 2A). PhII titers were greater than PhI titers for 11.2% (10/89) of the seropositive goats (Table 1). In 2019, seropositivity of the herd was 84.3% (113/134). The observed effect size for the proportion of seropositive goats between 2018 and 2019 was too low to determine significance with the available herd size (*p* = 1.00; power = 0.212, actual alpha = 0.034). PhII titers were greater than PhI titers for 5.3% (6/113) of the seropositive goats (*p* = 0.56; power = 0.357, actual alpha = 0.028, relative to 2018) (Supplementary Table S1). Herd seropositivity was 64.5% (20/31) in 2022, which decreased significantly relative to 2018 (*p* = 0.013; power = 0.860, actual alpha = 0.036) but not 2019 (*p* = 0.063; power = 0.691, actual alpha = 0.035). PhII titers were greater than PhI titers for 90.0% (18/20) of the seropositive goats on farm A, which was significantly increased relative to 2018 (*p* < 0.0001; power = 1.00, actual alpha = 0.0072) and 2019 (*p* < 0.0001; power = 1.00, actual alpha = 0.0039) (Supplementary Table S1).

Welch’s one-way ANOVA revealed a statistically significant difference in the geometric mean titer (GMT) between the study periods for PhI (*F* (2.00, 75.05) = [35.22], *p* < 0.0001) and PhII (*F* (2.00, 81.11) = [7.52], *p* = 0.001). In 2018, the GMT was 3,952 (range: <16–131,072) and 626 (range: 16–16,384) for PhI and PhII, respectively (Figure 2B). In 2019, the GMTs did not change significantly relative to 2018 with 2,277 (range: <16–262,144, *p* = 0.273) and 389 (range: <16–32,768, *p* = 0.1025) for PhI and PhII, respectively. In 2022, the GMT decreased significantly relative to 2018 with 100 (range: <16–262,144, *p* < 0.0001) and 175 (range: <16–32,768, *p* = 0.0012) for PhI and PhII, respectively. No statistically significant difference was found for GMT in 2022 relative to 2019 for PhII (*p* = 0.0580); however, PhI GMT was significantly different relative to 2019 (*p* < 0.0001).

**Figure 3:**
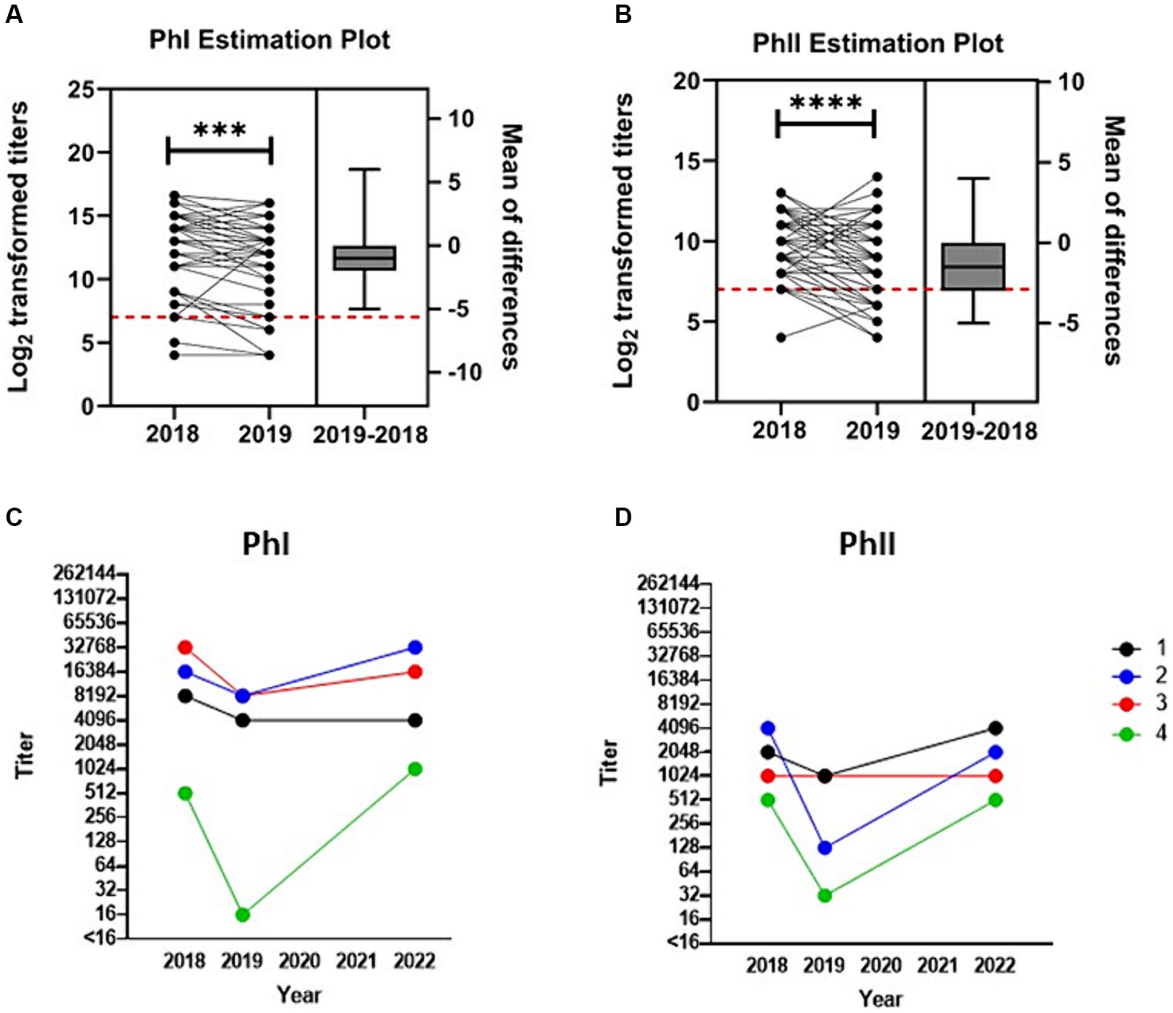
Anti-*C. burnetii* titers over time post-abortion storm. Transformed (log_2_) antibody titers against *C. burnetii* **(A)** PhI and **(B)** PhII are paired and plotted on the left y-axis for the 58 seropositive goats that were available for testing in both 2018 and 2019. One goat whose PhI titer fell to <16 in 2019 is not included in the PhI analysis. Titers ≥128 (red dashed line) are considered positive. Box and whiskers plot (± range) of the difference between the means for each pair are plotted on the right y-axis. ***p < 0.001, ****p < 0.0001 as determined by paired *t*-test. Serial testing for anti-*C. burnetii* antibody titers against **(C)** PhI and **(D)** PhII for the four goats tested across all periods.

Serological analysis was conducted on paired serum samples from 61 goats across both 2018 and 2019 (Figures 3A,B). GMT of this cohort was 5,043 (range: 16–131,072) and 881 (range: 16–8,192) for PhI and PhII, respectively. By 2019, titers decreased significantly relative to 2018 with GMT of 2,983 (range: <16–65,536, *p* = 0.0002) for PhI and 397 (range: 16–16,384, *p* < 0.0001) for PhII. Interestingly, three of these goats were seronegative at both samplings (Supplementary Table S1). Among the goats tested across both 2018 and 2019 that were seropositive in 2018, 5.2% (3/58) had PhII titers greater than PhI by 2019, which was significantly less than the proportion of seropositive goats in 2022 with PhII titers > PhI (*p* < 0.0001; power = 1.00, actual alpha = 0.007). Four goats were available for serological analysis across all samplings (Figures 3C,D). In 2018, the GMT of the four goats was 6,889 (range: 512–32,768) and 1,448 (range: 512–4,096) for PhI and PhII, respectively. In 2019, GMT for PhI was 1,448 (range: 16–8,192) and PhII was 256 (range: 32–1,024). In 2022, GMT for PhI was 6,889 (range: 1,024–32,768) and PhII was 1,448 (range: 512–4,096).

### Environmental burden of *C. burnetii* on the ranch of a naturally infected goat herd across four kidding seasons post-abortion storm

**Figure 4:**
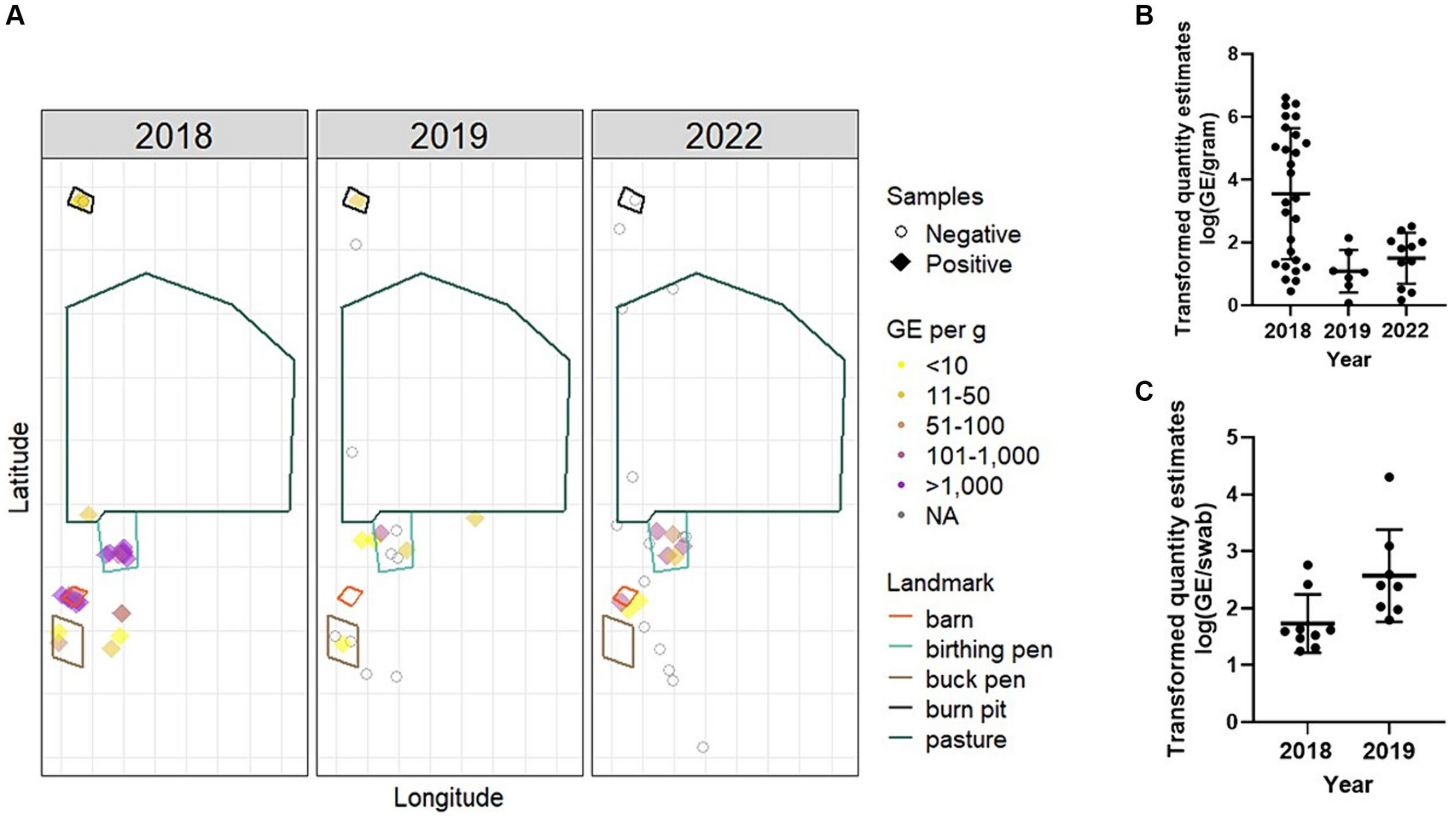
Environmental burden and spatial distribution of *C. burnetii* following an abortion event. Environmental samples were collected from various locations around the ranch. **(A)** Bulk samples were mapped by longitude and latitude and relevant geographic features from satellite imagery were overlayed. Negative samples are displayed as open circles. Positive samples are colored based on GE per gram of soil. One negative sample collected in 2019 from the boundary of the property is not displayed for better visualization of the remaining data points. Samples were analyzed by *IS1111* PCR for the presence of *C. burnetii* DNA. Data from positive **(B)** bulk soil and **(C)** environmental swabs are presented as the transformed (log_10_) quantity estimates for individual positive samples along with the geometric mean (bold horizontal bar) and geometric standard deviation (error bars). No environmental samples were positive in 2022.

In 2018, 83.7% (36/43) of the total environmental samples collected from locations around the ranch were positive for *C. burnetii* DNA (Table 1; Figure 4A). These samples were made up of bulk soil and environmental swabs for which 96.4% (27/28) and 60.0% (9/15) were positive, respectively. The GM quantity of *C. burnetii* among positive samples was 3.5×10^3 (95% CI: 528.9–2.4×10^4) GE per gram of bulk soil and 53.8 (95% CI: 21.7–133.2) GE per swab (Figures 4B,C). The greatest quantity of *C. burnetii* DNA from a single sample (4.1×10^6 GE/g) was collected from an area used for nursing/kidding. Genotyping of this sample identified the outbreak strain as sequence type 8. A total of 92.3% (24/26) of the samples from locations used for nursing/kidding were positive with a GM quantity of 1.1×10^5 (95% CI: 2.4×10^4–4.6×10^5) GE/g within the bulk soil (Table 1; Figure 4A). *C. burnetii* DNA was also identified in goat enclosure areas (4/5), on ranch equipment (2/5), at a burn pit utilized for placenta disposal (3/4) and in areas that were inaccessible to goats (3/3).

In 2019, total environmental positivity was 51.7% (15/29), which had decreased significantly relative to 2018 (*p* = 0.022; power = 0.855, actual alpha = 0.031). Positivity among bulk soil was 41.2% (7/17) with a GM quantity of 12.2 (95% CI: 2.9–51.4) GE/g. Among environmental swabs, 66.7% (8/12) were positive with a GM quantity of 372.8 (95% CI: 78.4–1.8×10^3). *C. burnetii* DNA was identified in goat enclosure areas (3/6), on ranch equipment (6/6), at a burn pit utilized for placenta disposal (1/1) and in areas that were inaccessible to goats (1/8).

In 2022, 28.6% (8/28) of the environmental samples were positive, which was significantly decreased relative to 2018 (*p* < 0.0001; power = 0.999, actual alpha = 0.032) but not 2019 (*p* = 0.319; power = 0.456, actual alpha = 0.028). No swabs (0/5) were positive in 2022. Among bulk soil, 34.7% (8/23) were positive with a GM quantity of 31.6 (95% CI: 9.0–111.0) GE/g. In 2022, *C. burnetii* DNA was identified in nursing/kidding areas (6/11), in goat enclosure areas (1/5), and in areas that were inaccessible to goats (1/9). No *C. burnetii* DNA was detected on ranch equipment (0/2) or in the burn pit (0/1) in 2022.

## Discussion

This study, which describes longitudinal surveillance of a *C. burnetii*-infected non-dairy goat herd following an abortion storm, found overall shedding of the herd was 14.4% in 2018, ~83–174 days post-outbreak. These findings align with data from experimentally infected pregnant does, which were shown to excrete *C. burnetii* at least 140 days post-infection (2). As shedding is highest immediately following parturition, the 2018 sampling likely underrepresents the true shedding that occurred during the abortion event. Despite the lengthy interval between sampling and parturition, the estimated quantity of *C. burnetii* in the vaginal mucus of one goat was 17,000 GE/swab; however, shedding varied markedly within the herd. For goats, shedding in vaginal secretions can be intermittent; therefore, it is not surprising that 89% of the herd were seropositive, suggesting more widespread infections than PCR analysis would suggest. Thus, approaches to *C. burnetii* surveillance among goat herds should incorporate both PCR and serology, which is supported by other caprine Q fever outbreak investigations (47, 48).

Throughout the study, no does were found to be shedding *C. burnetii* in the milk and vaginal secretions concurrently. In 2022, shedding in the herd was only detected in milk, which was likely influenced by the small sample size available for testing; however, it does suggest that strategies for determining herd shedding status should incorporate multiple sample types. Following an outbreak in Washington, shedding of *C. burnetii* in the milk occurred more frequently than in feces or vaginal fluids (49). The importance of shedding in milk has been described in other goat herds, which is not surprising given *C. burnetii* has been found in goat mammary gland epithelial cells (50, 51). This is contrary to previous studies, which demonstrated heavier and prolonged shedding via the vaginal or fecal route relative to milk (2, 19). Reasons for the conflicting findings are unclear but support the notion that analysis of multiple shedding routes should be considered when determining the *C. burnetii* status of a herd.

Goats experimentally infected with *C. burnetii* develop IgG PhII antibodies rapidly between 2 and 4 weeks post-infection (pi), which then increase slowly until 10 weeks pi (34, 35). IgG antibodies against the *C. burnetii* PhI antigen have been shown to develop later at 6 weeks pi, which then plateau at 9 weeks pi (34, 35). PhI antibodies persist following natural infection for at least two kidding seasons, whereas PhII antibodies typically decrease (17, 36). One study found that 60% of the herd were PhII seronegative by the second kidding season (17). In this study, PhI GMT among the goats available for testing in both kidding seasons decreased 1.7-fold, while PhII GMT decreased 2.2-fold. Given that PhI titers persist over longer periods relative to PhII, we categorized new infections as those where PhII > PhI. Of the seropositive goats, 11.2% had PhII antibody titers higher than phase I, 5 months post-outbreak, suggesting that new infections were continuing to some degree. In 2022, herd size had been reduced by approximately 78% relative to 2018 and the majority of goats present were either born or purchased post-abortion storm. At this time, 90.0% of the seropositive goats had antibody titers indicative of new infections, which is in stark contrast to the 11.2% in 2018 and 5.3% in 2019 and suggests that most of the herd had been recently infected. Despite the serological evidence of new infections and the finding that shedding was still occurring in 2022, the operation experienced no further *C. burnetii*-dependent abortions after the 2018 parturition period. This highlights the need for long-term monitoring of *C. burnetii*-infected herds even in the absence of symptoms. This also underscores the need for improved and accessible mitigation strategies post-outbreak.

Five-months post-outbreak, *C. burnetii* was widespread in the environment with 96.4% positive bulk samples with a mean of three thousand *C. burnetii* in 1 gram of soil and reached as high as four million bacteria per gram in the birthing pens. A study of naturally infected sheep found that *C. burnetii* did not persist in the soil beyond 1 month post-lambing season; rather, it was reintroduced during subsequent parturitions (52). In the laboratory setting, *C. burnetii* is reported to remain viable at least 20 days in soil and was found to have a decay rate of 4% per min when tested at 30% relative humidity in sunlight. (53, 54). This suggests that *C. burnetii* present in the environment in 2019 and 2022 was reintroduced by the herd, which is supported by the evidence of new infections and continued shedding across the study period. In 2019, 12 months post-outbreak, the overall *C. burnetii* environmental burden had decreased to 51.7 and 28.6% in 2022, 49 months post-outbreak. Despite continued infections in the herd and shedding in 2019 and 2022, environmental contamination was not sustained at high levels beyond the abortion event.

Shedding in the 2019 and 2022 sampling periods occurred despite the annual administration of CTC. This aligns with previous studies in ruminant livestock, which have found that antibiotic administration in goats is insufficient to eliminate *C. burnetii* (18, 28, 47, 55). Vaccination of ruminant herds (for both prevention and outbreak control) has been shown to reduce reproductive losses, shedding, and therefore risk to humans (22–29). However, vaccination is not available in some countries and thus could not be utilized by the operation (27, 28). Culling of animals has been utilized in the past in conjunction with vaccination as a means of controlling *C. burnetii*-infected herds (18, 56, 57); however, it is not recommended by the National Association of State Public Health Veterinarians and the National Assembly of State Animal Health Officials (58). Larger herds have been identified as a risk factor for *C. burnetii* in previous studies, and one study found that positivity increased with the number of goats per acre (59–62). Although the reasons for the reduction in herd size and the selection of goats for removal in this case were unrelated to the outbreak, the herd was reduced by ~78% by 2022. Despite this and other management practices such as routine disposal of placentas by burning, shedding and environment contamination were observed 4 years post-outbreak. Further highlighting the need for improved and widely accessible *C. burnetii* mitigation strategies on goat operations.

Limitations of this study include analysis of a working ranch for which the investigators had no operational oversight. Due to the observational nature of the study, it was not possible to control for certain experimental parameters including sample collection timing, control measures, herd additions/losses, or determination of pre-outbreak *C. burnetii* status. As shedding of *C. burnetii* is highest immediately following kidding, the 5-month period between the outbreak and initial sampling likely led to an underrepresentation of shedding. Although ~83% of the herd was tested during the initial sampling, it is possible that the analysis did not capture the true burden of disease. However, to decrease potential bias, testing was conducted on all goat types present at the time regardless of age, sex, breeding status, or kidding outcome. The use of different extraction kits across the study period due to discontinuation by manufacturers could influence comparisons; however, total DNA yield and purity did not differ significantly by kit (data not shown). Variability in the percentage of samples taken from different areas across the operation from year to year may lead to over or under-representation of some areas. Fecal swabs are frequently analyzed during *C. burnetii* investigations; however, the interpretation of the sample type has limitations (2, 13, 63). Experimental oral infection in mice suggests that *C. burnetii* may replicate in the stomach; however, it is not clear whether *C. burnetii* detected on fecal swabs represents active infections or simply expelled bacteria following ingestion from the contaminated environment (64). Finally, the presence of other *C. burnetii* strains on the ranch with different *IS1111* copy numbers could influence the quantity estimates.

In conclusion, *C. burnetii*-infected goats can be devastating to an operation and public health. This study found shedding and environmental contamination on a non-dairy goat operation four kidding seasons post-abortion storm. Regardless of whether *C. burnetii* was perpetuated from the abortion event, reintroduced, or both, this study highlights that goat producers should be aware of the potential for the long-term presence of *C. burnetii* on the operation. Control measures for the prevention and eradication of *C. burnetii* in livestock are limited, and vaccination of animals is currently the most successful strategy (28). Vaccination of ruminant herds (for both prevention and outbreak control) has been shown to reduce reproductive losses, shedding, and therefore risk to humans (22–29). However, vaccination is not available in some countries and boosters are recommended every 280 days (27, 28). A better understanding of management practices that can improve outcomes for infected herds, particularly in areas without access to vaccines against *C. burnetii*, is needed to better protect operators and the public from this challenging organism.

## Data Availability

The original contributions presented in the study are included in the article/Supplementary material, further inquiries can be directed to the corresponding author.
